# Sphingolipid metabolism in the development and progression of cancer: one cancer's help is another's hindrance

**DOI:** 10.1002/1878-0261.13063

**Published:** 2021-07-29

**Authors:** Antonia Piazzesi, Sumaiya Yasmeen Afsar, Gerhild van Echten‐Deckert

**Affiliations:** ^1^ LIMES Institute for Membrane Biology and Lipid Biochemistry University of Bonn Germany

**Keywords:** cancer, ceramide, gangliosides, sphingolipid, sphingosine kinase, sphingosine‐1‐phosphate

## Abstract

Cancer development is a multistep process in which cells must overcome a series of obstacles before they can become fully developed tumors. First, cells must develop the ability to proliferate unchecked. Once this is accomplished, they must be able to invade the neighboring tissue, as well as provide themselves with oxygen and nutrients. Finally, they must acquire the ability to detach from the newly formed mass in order to spread to other tissues, all the while evading an immune system that is primed for their destruction. Furthermore, increased levels of inflammation have been shown to be linked to the development of cancer, with sites of chronic inflammation being a common component of tumorigenic microenvironments. In this Review, we give an overview of the impact of sphingolipid metabolism in cancers, from initiation to metastatic dissemination, as well as discussing immune responses and resistance to treatments. We explore how sphingolipids can either help or hinder the progression of cells from a healthy phenotype to a cancerous one.

## Graphical abstract

This review gives an overview on the impact of sphingolipid metabolism in various cancer types, from initiation to metastatic dissemination, as well as on immune response and resistance to cancer treatments. It explores how sphingolipids can either help or hinder the progression of cells from a healthy to a cancerous phenotype, and discusses the therapeutic potential of targeting sphingolipid metabolism for the treatment of patients with cancer.

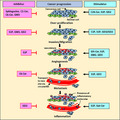

**Abbreviations**
C1P: ceramide‐1‐phosphate
CTCs: circulating tumor cells
ER: endoplasmic reticulum
GalCer: galactosylceramide
GlcCer: glucosylceramide
GSL: glycosphingolipid
S1P: sphingosine‐1‐phosphate
SK: sphingosine kinase
SPL: S1P‐lyase
TGN: trans‐Golgi network

## 1 Introduction

**Fig. 1:**
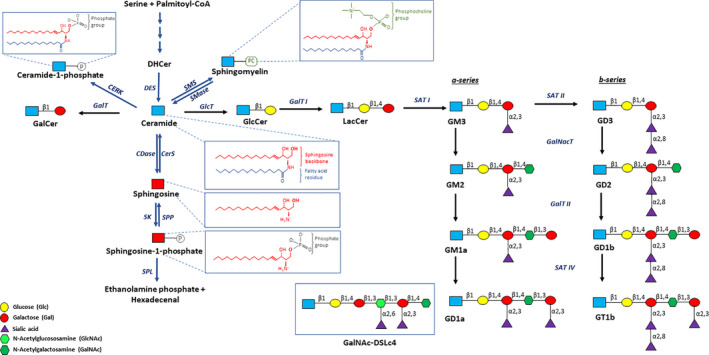
Schematic of ceramide metabolism including the biosynthesis of major mammalian gangliosides. All glycosylation steps except the initial glucosylation of ceramide are mapped to the luminal face of Golgi/TGN membranes. GM3 and GD3 are formed by the action of specific sialyltransferases (SAT I and II), whereas rather unspecific glycosyltransferases catalyze the stepwise addition of a definite sugar as indicated. Inserts: Structures of ceramide, sphingosine, sphingosine‐1‐phosphate, and GalNAc‐DSLc4 (*N*‐acetylgalactosaminyl‐disialyl lactotetraosyl), a hybrid structure between the ganglio‐ and the lacto‐series. Abbreviations used are (a) for lipids: DHCer, dihydroceramide; Gal, d‐galactose; GalNAc, *N*‐Acetyl‐d‐galactosamine; Glc, d‐glucose; the terminology used for gangliosides is that of Svennerholm [210]; (b) for enzymes: CDase, ceramidase (5 human isoforms, acid, neutral, alkaline 1,2,3); CERK, ceramide kinase; CerS, ceramide synthases (6 isoenzymes); DES, dihydroceramide desaturase; GlcT, ceramide glucosyltransferase; GalT I, galactosyltransferase I (lactosyl synthase); GalT II, galactosyltransferase II (GM1a/GD1b synthase); GalNAcT, *N*‐Acetyl‐d‐galactosaminyltransferase (GM2/GD2 synthase); SAT I, sialyltransferase I (GM3 synthase); SAT II, sialyltransferase II (GD3 synthase); SAT IV, sialyltransferase IV (GD1a/GT1b synthase); SK, sphingosine kinases (two isoforms SK1 and SK2); SMS, sphingomyelin synthase; SMase, sphingomyelinases (two main isoforms, acid aSMase and neutral nSMase); SPL, sphingosine‐1‐phosphate lyase; SPP, S1P phosphatases (two known isoforms SPP1 and SPP2). Note that all biosynthetic steps (black arrows) are reversible.

Discovered in the 1870s and named for their unique and enigmatic structure, sphingolipids [1] consist of a fatty acid residue bound to a sphingosine backbone, thus forming ceramide (Fig. 1), their membrane anchor that can show diverse characteristics [2]. The hydroxyl group of carbon atom 1 can be further modified by the addition of either a phosphate group, forming ceramide‐1‐phosphate (C1P), a phosphocholine group, yielding sphingomyelin, or a series of sugar residues, generating quite diverse glycosphingolipids (GSLs) (Fig. 1).

**Fig. 2:**
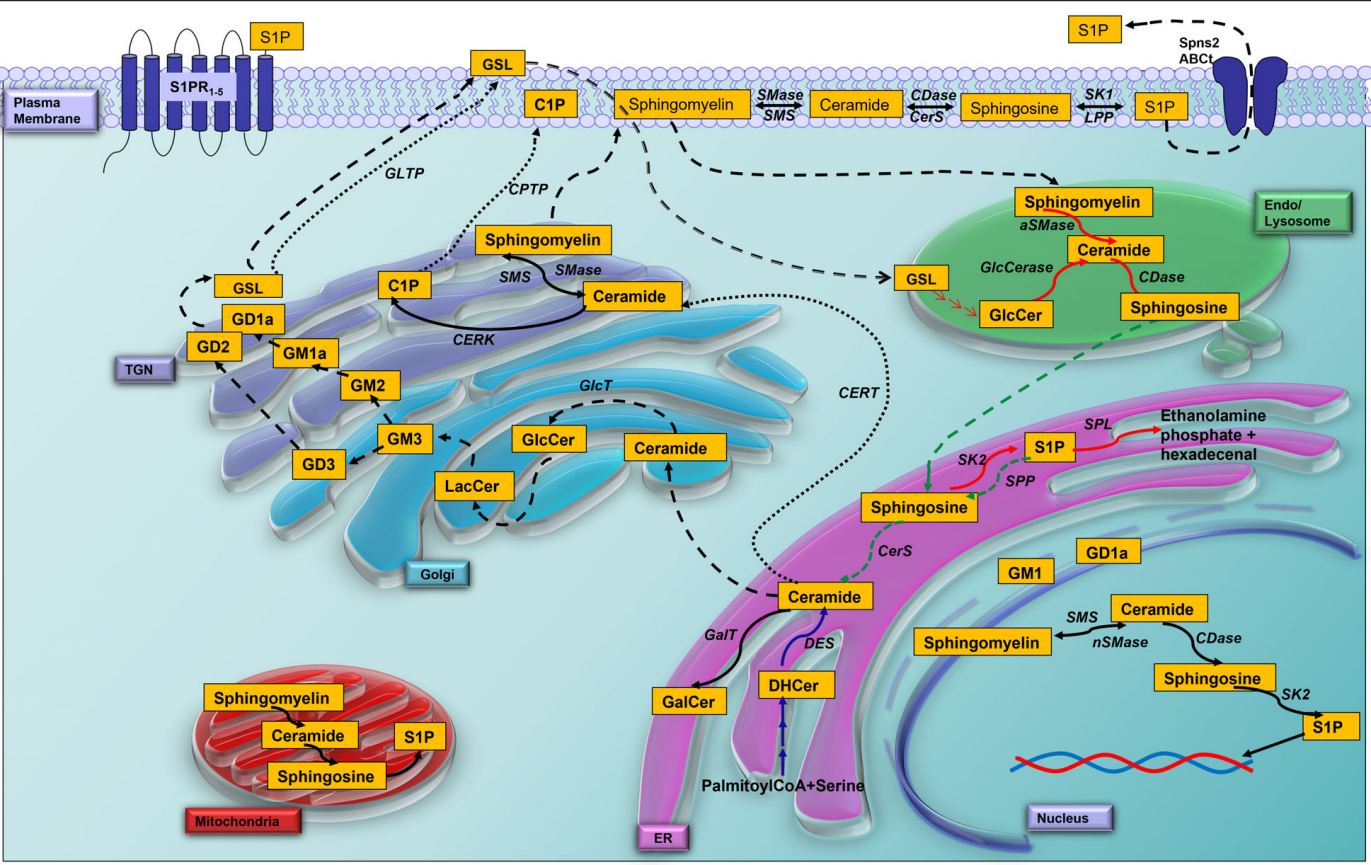
Schematic of prevalent locations and metabolic pathways of cellular sphingolipids. Ceramide is biosynthesized *de novo* in the endoplasmic reticulum (ER, purple, blue arrows). It is then translocated via the ceramide transport protein (CERT, black dotted arrows) to the site of sphingomyelin and ceramide‐1‐phosphate (C1P) formation in the trans‐Golgi network (TGN, purple) or via vesicular exocytotic membrane flow (black dashed arrows) to the site of glycosylation to glucosylceramide (GlcCer) in the Golgi compartment (blue) and more complex glycosphingolipids (GSL), including gangliosides (black dashed arrows). Note that only GM3 and GD3, the precursors of a‐ and b‐series of gangliosides, are generated in the Golgi, whereas all the following glycosylation steps are located to the TGN (lilac). Degradation of sphingolipids down to sphingosine occurs mainly in the lysosomal compartment (red arrows). Further metabolization of sphingosine is located to the ER, where it is first phosphorylated to sphingosine‐1‐phosphate (S1P) and then cleaved into ethanolamine phosphate and hexadecenal. Alternatively, S1P can be dephosphorylated back to sphingosine and further recycled to ceramide and all other sphingolipids via the salvage pathway (green dashed arrows). S1P generated in the plasma membrane by SK1 can be transported into the extracellular milieu via ATP‐binding cassette transporters (ABCt) or spinster homolog 2 (SPNS2) where it acts as a ligand of five G‐protein coupled receptors (S1PR1‐5). Transport of (glyco)sphingolipids occurs via vesicles (dashed black arrows) or transport proteins (dotted arrows). Note that sphingolipids are metabolized also in the plasma membrane, mitochondria, and nuclei. Abbreviations used are CPTP, C1P transport protein; GlcCerase, glucocerebrosidase; GLTP, glycolipid transport protein; and LPP, lipid phosphate phosphatase. All other abbreviations as in legend to Fig. 1.

Ceramides are synthesized in the endoplasmic reticulum (ER) and can then be transported via the Golgi apparatus to the cell membrane as structural components of the lipid bilayer, to the nucleus, or to the mitochondria, where they function primarily as signaling molecules [3]. Apart from being sphingolipids in their own right, ceramides are also metabolic intermediates for other members of the sphingolipid family. They can be transported to the Golgi, where the addition of a phosphocholine group to their C1‐hydroxyl group yields sphingomyelins, which represent the majority of sphingolipids in the human body largely due to their structural role in cell membranes, particularly in the myelin sheath surrounding axons [4]. As illustrated in Fig. 2, glycosphingolipids (GSLs) are synthesized when ceramide is transported via vesicular membrane flow from the ER to its site of glucosylation in the Golgi compartment, while galactosylation of ceramide occurs in the ER. Further sugar residues are attached stepwise in the Golgi and in the trans‐Golgi network (TGN). GSLs can be further subdivided into either cerebrosides or gangliosides. Cerebrosides, including GlcCer and GalCer, have only one sugar residue on the C1‐hydroxyl group of ceramide and, as their name suggests, are largely found in the brain. Gangliosides are GSLs with one or more sialic acid residues in their sugar chain and are also abundant in the brain, forming characteristic patterns in neuronal membranes [4]. The complexity of gangliosides depends on the number of sugars and the number of sialic acids bound to a defined galactose residue of the sugar chain on the C1‐hydroxyl group of their ceramide membrane anchor (Fig. 1). Thus, we can further differentiate gangliosides with one (GM; monosialo‐), two (GD; disialo‐), three (GT; trisialo‐), or up to five (GP; pentasialo‐) sialic acid residues linked either to the inner or to the outer galactose of the sugar chain or to one of these already attached sialic acid residues. Gangliosides are generated by stepwise glycosylation of ceramide in different Golgi stacks and this process is intimately coupled with the exocytotic vesicular membrane flow [5]. GM3 and GD3, which are the precursors of a‐ and b‐series gangliosides, respectively (Fig. 1), are formed in the proximal Golgi compartment, whereas more complex gangliosides are generated more distally in the TGN (Fig. 2).

Gangliosides are often found to be concentrated in lipid rafts in the cellular plasma membrane, where they play an important role in cell adhesion and signal transduction [6]. Exogenously applied gangliosides are rapidly incorporated into the plasma membrane and play key roles in cell–cell interaction, differentiation, and oncogenesis [7, 8]. Constitutive degradation of sphingolipids down to sphingosine occurs in the endosomal/lysosomal compartment [9]. Breakdown of sphingosine follows after its phosphorylation to sphingosine‐1‐phosphate (S1P) and cleavage into phosphoethanolamine and hexadecenal in the ER (Fig. 2). Alternatively, S1P can be recycled after dephosphorylation to sphingosine followed by N‐acylation to ceramide and generation of all other more complex sphingolipids by the salvage pathway (Fig. 2).

While they were initially thought to be little more than lipids integral to the structure and maintenance of the plasma membrane, it was later discovered that sphingolipids are also bioactive molecules, with an active role in cell signaling and the regulation of a multitude of different processes [10]. It should come as no surprise, therefore, that sphingolipids have also been implicated in cancer, the development of which involves a number of different cellular processes. In order for a healthy cell to become a malignant tumor, it must overcome a number of obstacles set in place by the organism to protect against such diseases. First, the cell must acquire the ability to proliferate, both by changing its metabolism to facilitate cell division and by bypassing the usual blockades that exist to prevent unchecked growth []. Second, it must be able to invade the neighboring tissue, changing its morphology and migratory capabilities by co‐opting signaling programs usually restricted to embryonic development and immune cell trafficking [12]. Once the mass of proliferating cells reaches a certain size, it must then be able to form additional blood vessels so as to bring oxygen and nutrients to its center, lest it starve from the inside out [13]. Finally, in order to become metastatic, the cells must survive the usually lethal process of detaching from the extracellular matrix so as to spread throughout the body, all the while evading an immune system on patrol for transformed cells.

We have chosen to represent the progression of cancer as a linear, stepwise process for the purpose of clarity. It is, however, important to note that this is a simplification of the biological reality. Tumor angiogenesis, for example, does not only occur in order to supply oxygen and nutrients to the growing mass, but it is also known to enable tumor invasion itself [14], while its suppression can halt invasion without suppressing proliferation [15]. Also, the immune system is able to target cancerous cells before they detach and circulate in the organism, and thus, both immune cell evasion and the response to inflammation are processes that occur throughout the life cycle of the tumorigenic cell [16, 17]. The fact that there are almost no simple, linear relationships of cause and effect in biology represents one of the greatest challenges in the field. In this Review, we attempt to elucidate how bioactive sphingolipids can fit into this complex web. We will discuss the steps that a healthy cell undertakes to become cancerous and how sphingolipids can either help it along its journey or block its path.

## 2 Step 1: Proliferation

In order for a cell to become cancerous, it must first overcome the systems set in place to prevent it from overproliferating, such as cell cycle arrest and apoptosis [18]. As a regulator of apoptosis, ceramide is one of those molecules that cancerous cells need to circumvent in order to achieve the necessary proliferation, which is probably why many cancerous cell lines have been found to undertake its metabolic transformation/degradation [19]. For these reasons, restoring ceramide levels in cancerous cells has been a focus of study for decades.

Direct treatment with either sphingosine, ceramide, or ceramide analogs was found to inhibit cell proliferation and induce apoptosis in *in vitro* models of colon cancer [20, 21, 22], breast cancer [23], nasopharyngeal carcinoma [24], and pancreatic cancer, especially when used in conjunction with chemotherapeutic drugs [25, 26] (Table 1). Furthermore, multiple studies have found that some chemotherapeutic agents themselves can block cell proliferation and induce apoptosis by increasing ceramide production in many different cellular models of cancer, including breast [27, 28, 29, 30] and prostate [31, 32, 33] cancer, as well as in HeLa and human kidney carcinoma cells [34] (Table 1).

**Table 1:** Representative studies on the dual role of sphingolipids in cancer. ApcMin, mouse model for intestinal tumorigenesis; Bcl‐2, B‐cell lymphoma 2; Bcl‐xL, B‐cell lymphoma‐extra‐large; *Brms1*, gene encoding breast carcinoma metastasis suppressor 1; CD44, cluster determinant 44; Cer, ceramide; CerS, ceramide synthase; CSCs, cancer stem cells; EGFR, epidermal growth factor receptor; ER, endoplasmic reticulum; ERK1/2, extracellular signal‐regulated kinases 1/2; FAK, focal adhesion kinase; HNSCCs, human head and neck squamous cell carcinomas; HOS, human osteosarcoma; HOSE, human ovarian surface epithelial; HUVECs, human umbilical cord vein endothelial cells; ICAM‐1, intercellular adhesion molecule 1; IFN‐γ, interferon‐gamma; IL, interleukin; NFκB, nuclear factor kappa‐light‐chain‐enhancer of activated B cells; PI3K, phosphoinositide 3‐kinase; PKCζ, protein kinase C zeta; PLCβ1, phospholipase C beta 1; Rac1, ras‐related C3 botulinum toxin substrate 1; Rb, retinoblastoma protein; SCLCs, small‐cell lung cancers; SNAI2, Snail family transcriptional repressor 2; *Spns2*, S1P transporter spinster homologue 2; TGF‐β1, transforming growth factor‐beta1; TRAX, translin‐associated factor X; UM‐SCC‐22A cells, squamous cell carcinoma of hypopharynx; mUOG1/mLAG1, mammalian upstream regulator of growth and differentiation factor 1/mouse homologue of longevity assurance gene 1; VEGF, vascular endothelial growth factor.

Cancer progression	Sphingolipid	Molecular mechanism involved	Sphingolipid manipulation strategy	Effect on cancer	Biological material	Reference
Cellular overproliferation	C_2_‐Cer/sphingoid bases	Cell cycle arrest in G2 or G1 phase; Upregulation of p27	Exogenously applied	Inhibition	Human colon cancer cell lines; HNSCC cells	[20, 21, 24]
	C_2_‐ C_6_‐ C_18_‐Cer	Release of cytochrome *c*/caspase activation	Exogenously applied; Ceramidase inhibition	Inhibition	SW403 colon cancer cell line	[22]
	C_18_‐Cer	Mitochondrial death pathway, inhibition of telomerase activity	Overexpression of mLAG1/mUOG1	Inhibition	UM‐SCC22A cells	[35]
	C_16_‐Cer	Prevents ER‐stress‐mediated apoptosis	Induced CerS6 expression	Promotion	Diverse HNSCC cell lines	[36]
	C_24_‐Cer	Not aim of this study	Overexpression of CerS2	Promotion	Human breast and colon cancer cell lines	[37]
	Sphingosine	Cell cycle arrest at G1/S by reduced expression of CDK4 and diminished Rb phosphorylation	Exogenously applied *in vitro;* SK1 deficiency *in vivo*	Inhibition	Rat intestinal epithelial cells; SK1‐deficient ApcMin mice	[58]
		Increased expression of cell cycle inhibitors	p53‐dependent downregulation of SK1	Inhibition	Several cancer cell lines; p53‐deficient MEFs	[54]
	S1P	Mobilization of calcium, ERK1/2 activation	Estrogen‐induced stimulation of SK1; Overexpression of SK1	Promotion	Diverse human breast cancer cell lines	[39]
		S1P_3_‐dependent transactivation of EGFR				[40]
		Activation of NFκB‐p65 and increased cyclin D1 expression		Promotion	Human breast epithelial cells	38
		S1P/S1PR_3_‐mediated Notch activation	Exogenously applied		CSCs; nude mice	[43]
		Upregulation of anti‐apoptotic proteins Bcl‐2 and Bcl‐xL	SPL deficiency	Promotion	*Sgpl1* ^(−/−)^ MEFs; nude mice	[60]
	GM3	Decreased phosphorylation of EGFR and reduced cell adhesion	Exogenously applied *in vitro* and *in vivo*	Inhibition	Human bladder cancer cell lines/mouse model	[89]
	GD3/GD2	Stimulates activation of mitogen‐activated protein kinases	Overexpression of GD3 synthase	Promotion	SCLC cell lines; SCLCs	[151]
Cell migration/invasion	C1P	Gi protein coupled receptor‐mediated activation of ERK1/2, PI3K/AKT signaling; Activation of RhoA/ROCK1	Exogenously applied	Promotion	RAW264.7macrophages	[81]
					Human pancreatic cancer cell lines	[83]
	S1P	S1P/S1PR_1_‐mediated activation of Rac1/ PI3K signaling	Exogenously applied	Promotion	Wilms tumor	[65]
		S1P/S1PR_2,3_‐mediated increase of SNAI2 expression			Breast cancer cell lines	[73]
		S1P/S1PR_3_‐mediated activation of AKT signaling			Nasopharyngeal carcinoma cell lines	[75]
		S1P/S1PR_2_‐dependent enhancement of stress fibers		Inhibition	HOSE cell line	[78]
	GD3/GD2	Increased phosphorylation of p130Cas, paxillin and FAK	Induction of GD3 synthase	Promotion	Several HOS cell lines	[95]
	GM2	Activation of TGF‐β1 signaling	Modulation of glycolipid synthesis	Promotion	Pancreatic ductal adenocarcinoma cell lines	[93]
	GD3/GD2	Downregulation of ICAM‐1 expression, inhibition of AKT signaling	Overexpression of GD3 synthase	Inhibition	Breast cancer cell line	[98]
	GM3	Mediates formation of complex between CD9 and integrin receptors	Enhanced GM3 synthesis	Inhibition	C3H fibroblast 10T1/2 cells transformed with v‐Jun	[87]
Angiogenesis	Sphingomyelin‐derived Cer	Regulates exosomal angiogenic miRNA secretion (miR‐210)	Modulation of nSMase2	Promotion	Different breast cancer cell lines; HUVECs; Normal mammary epithelial cells	[117]
	S1P	S1P/S1PR_1/3_ ‐mediated secretion of angiogenic factors (VEGF, IL‐8, IL‐6)	Exogenously applied; Modulation of SK1and of S1PR_1/3_	Promotion	Ovarian cancer cells, tissue, mouse model	[106]
		S1P/S1PR_2_/G(12/13)/Rho‐dependent suppression of Rac1/AKT signaling	Modulation of S1PR_2_	Inhibition	Tumor isograft models, S1PR_2_‐deficient mice	[112]
	Globo‐H Cer	Ca^2+^ mobilization by binding TRAX and thus releasing and activating PLCβ1	Exogenously applied as microvesicles shed from breast cancer cells; subcutaneous injection	Promotion	HUVECs; Diverse breast cancer cell lines; Balb/C mice	[118]
Metastasis	C_6_‐Cer	Induction of anoikis by activation of caspase 3/7 and inhibition of CD44;	Exogenously applied as nanoliposomes	Inhibition	Human breast and pancreatic cancer and melanoma cells	[142]
		Activation of PI3K and PKCζ and hence reduction of integrin affinity				[143]
	S1P	Upregulation of the metastasis‐promoting gene *FSCN1* expression via activation of NFκB	Modulation of SK1	Promotion	Human and mouse cancer cells; Nude mice injected with human cancer cells	[136]
		Prevents activation of *Brms1* via modulation of S1PR_2_	Modulation of SK1 and of S1PR_2_; Tail vein injection of murine cancer cells	Promotion	Multiple cancer cells; WT and SK1^−/−^ mice	[137]
	GM2	Not aim of these studies	Modulation of GM2 expression using humanized anti‐GM2 antibodies	Promotion	GM2‐expressing SCLC multiple organ metastasis model	[129]
	GD2		Modulation of GD2 expression using a GD2 monoclonal antibody		Murine lymphoma EL4 cells injected into syngeneic C57BL/6 mice	[128]
Immune response	S1P	Lymphopenia and a higher percentage of effector T and natural killer cells in the lung and the liver	Depletion of *Spns2*	Inhibition	Several mouse models and cancer cell lines	[164]
	α‐GalCer	Activation of natural killer T cells and increase of IL‐12 p40 and IFN‐γ inducible protein 10 in serum	Intravenous injection of dendritic cells loaded with α‐GalCer	Inhibition	Patients with advanced cancer	[156]
	Gangliosides	Sialic acid‐dependent inhibition of mitogen‐ and antigen‐induced lymphocyte activation	Modulation of sialic acid in human leukemia gangliosides	Promotion	Human leukemia cells	[146]

Although these studies may seem promising, it is important to not lose sight of the fact that ceramides are actually a family of bioactive lipids, with different chain lengths and different roles in signaling and thus also different effects on the proliferative properties of cancer cells (Table 1). For example, in human head and neck squamous cell carcinoma (HNSCC) tumor tissue, only C_18_‐ceramide was found to be downregulated when compared to neighboring, healthy tissue [35]. In a follow‐up study on an *in vitro* model of HNSCC, C_18_‐ceramide was found to induce cell death, whereas C_16_‐ceramide actually protected the cells from apoptosis and enhanced tumor development [36]. In human breast cancer and colon cancer cell lines, on the other hand, increasing the levels of C_16_‐, C_18_‐, and C_20_‐ceramides via genetic manipulation all led to an inhibition of cell proliferation, whereas increasing the amount of C_24_‐ceramide actually enhanced proliferation [37], indicating that the effects of different ceramides can also be cancer cell line‐specific (Table 1).

Further complicating matters is the fact that ceramides are metabolic intermediates for other bioactive sphingolipids, which can then in turn influence the ability of the cell to proliferate unchecked. The lysis of ceramide by ceramidases and their subsequent phosphorylation by sphingosine kinases (SKs) leads to the production of S1P, which has its own active role in cell signaling. In a breast cancer cell line, the production of S1P via the overexpression of SK1 led to the overexpression of cyclin D1, thereby shortening the cell cycle and increasing cell proliferation [38]. It has also been shown that, in breast cancer cells, SK1 is activated by estrogen (E2), serving as a mediator of downstream signaling cascades such as calcium mobilization and ERK1/2 activation [39]. Moreover, release of S1P as a result of SK1 stimulation by E2 activates S1P receptor 3 (S1PR_3_) and transactivates epidermal growth factor receptor (EGFR) [40]. These two studies reveal the essential role of SK1/S1P as mediators of the growth‐promoting effect of estrogen in human breast cancer cells (Table 1). In a mouse model of colitis‐associated cancer (CAC), genetic inhibition of S1P degradation led to enhanced tumor formation [41]. Conversely, enhanced S1P degradation impeded cell proliferation and tumorigenesis in both mice with CAC [41] and human osteosarcoma cell lines [42]. Enhanced S1P production has also been found to enhance and expand the proliferative potential of cancer stem cell populations [43], including breast cancer [44], and esophageal adenocarcinoma stem cells [45], demonstrating the strong proproliferating properties of S1P. In this context, we also recommend an excellent recent review by Ng *et al*. [46].

Together, these results, as well as the involvement of SK1 in oncogenic H‐Ras‐mediated transformation [47], clearly document the tumorigenic properties of SK1. We want to emphasize that, although SK1 is not a traditional oncogene [48], its activity defines the amount of either the growth‐stimulating S1P or the pro‐apoptotic sphingosine and ceramide [49]. Indeed, SK1 is highly upregulated in many cancers, including breast cancer, colon cancer, head and neck cancer, and glioblastoma [50, 51, 52, 53]. It is therefore not surprising that numerous studies were undertaken to uncover the factors that regulate the expression and activity of SK1. One of the negative regulators of SK1 turned out to be the tumor‐suppression protein p53 [54], which is involved in more than 50% of all human tumors [55, 56, 57]. Downregulation or depletion of SK1 is often associated with an increase of its substrate, sphingosine, which was shown to inhibit cell proliferation by affecting cell cycle progression [58]. This finding suggests that SK1 activity, which defines cellular amounts of S1P and sphingosine, influences tumorigenesis in general and intestinal cancer in particular (Table 1). We therefore recommend an excellent review about the complex system of transcription factors, cytokines, and micro‐RNAs involved in the modulation of SK1 expression [59].

Apart from SK1, there is also S1P‐lyase (SPL), the enzyme that irreversibly cleaves S1P, which can affect cell proliferation. SPL deficiency was shown to increase cell proliferation, anchorage‐independent cell growth, and tumor formation in nude mice [60]. These effects were explained by an upregulation of Bcl‐2 proteins in cells lacking SPL. Moreover, disruption of SPL in mouse embryonic fibroblasts (MEFs) conferred resistance to chemotherapeutic drugs [60] (Table 1). In addition, SPL deficiency considerably affected sphingolipid metabolism in MEFs. Thus, the formation of metabolic precursors such as ceramide, GlcCer, and GM3 was substantially elevated at the expense of more complex gangliosides, levels of which in turn dropped significantly [61]. The fact that the increased amounts of complex sphingolipid precursors are generated in the ER (ceramide) and in the Golgi apparatus (sphingomyelin, GlcCer, GM3), whereas the reduced amounts of complex GSL are made in the TGN (GM2, GM1, GD1a), points to a potential transport deficit in these cells. Together, these results illustrate the complexity and often the duality of the relationship between sphingolipid metabolism and cancer development. Thus, the addition of a sugar group to the C1‐hydroxyl group of ceramide by glucosylceramide synthase (ceramide glucosyltransferase; GlcT) can add another final layer of complication to cancer cell signaling. The production of GlcCer (i.e., a subset of cerebrosides) can not only confer resistance to chemotherapeutic drugs [62], but it can also protect cells against the pro‐apoptotic effects of ceramide itself [63]. Taken together, these studies show that, when trying to affect cancer cell growth, it is not enough to attempt to manipulate the levels of any one single sphingolipid. Rather, it is a metabolic balance of different sphingolipids, which can either work together or against each other to determine whether cancerous cells will proliferate or die.

## 3 Step 2: Invasion

Once cells begin to proliferate, they then need to gain the ability to invade neighboring tissue in order for the mass to grow. This involves a number of structural and physiological changes that confer the abilities of migration and remodeling that are usually restricted to either embryonic development or immune cell trafficking [12]. Given that S1P is integrally involved in many aspects of both embryogenesis and immune cell migration, it is no surprise that cancer cells have been found to co‐opt this molecule for the purposes of gaining the ability of invasion.

Since the 1990s, S1P has been known to be a central mediator of cell migration via intercellular cell signaling [64]. In the immune system, S1P is synthesized and exported to the extracellular matrix, where it binds S1P receptors (Fig. 2) and triggers the signaling cascades necessary for the appropriate physiological changes to occur for immune cell migration [64]. Numerous different types of cancer have been found to take advantage of this intercellular communication via S1P in order to acquire the migratory capabilities necessary for invasion, including Wilms renal tumors [65], human glioblastoma cells [66], prostate cancer [67, 68], hepatocellular carcinoma [69], thyroid cancer [70], breast cancer [71, 72, 73], pediatric alveolar rhabdomyosarcoma [74], nasopharyngeal carcinoma [75], and ovarian cancer [76, 77]. While this may seem like a virtually universal role for S1P in promoting cell migration by intercellular communication, some studies have actually suggested that this, too, could be a cell type‐specific effect. One study found that, although S1P promoted the invasion of epithelial ovarian cancer cells, it actually inhibited the cell migration of immortalized human ovarian epithelial cells [78] (Table 1). In an organotypic model of prostate cancer, S1P supplementation restored acinar structures and blocked the invasiveness of these 3D organotypic cultures [79]. Interestingly, both of these studies involved the cotreatment of S1P with lysophosphatidic acid (LPA), a serum phospholipid which is also associated with the promotion of cell migration and tumor invasion [80]. Taken together, these two studies seem to suggest that the presence of two bioactive lipids, which individually promote cell migration and invasion, can actually have the opposite effect when present in the extracellular milieu at the same time. It is studies like these that further elucidate why it is so important to never lose sight of the larger biological context before drawing conclusions on the signaling capabilities of any one molecule.

Similarly, C1P, generated from ceramide by ceramide kinases (Fig. 2), represents another bioactive sphingolipid with its own role in stimulating cell migration, particularly in macrophages [81]. Given this ability, C1P often acts either alone or in concert with S1P to stimulate cancer cell invasion, such as in pancreatic cancer cells [82, 83] and vascular endothelial cells [84].

The ability of the cell to migrate and invade neighboring tissue is necessarily accompanied by changes in the shape of the cell. As structural components of the cell membrane, therefore, it stands to reason that gangliosides also have their role to play in the invasive potential of cancerous cells, which is why aberrant glycosylation of GSLs has long been known to define tumor malignancy [7]. Furthermore, the chemical composition of different gangliosides gives them diverse structural properties, which is why their relative abundance in the cell membrane can have different effects on the ability of cancerous cells to invade neighboring tissue.

Intriguingly, variations in the expression of a certain ganglioside as well as modifications of its sialic acid residues can induce either procancerous or anticancerous effects. Thus, overexpression of GM3 was shown to reduce invasiveness and hence malignancy of murine bladder tumor cells [85], whereas silencing of GM3 synthase suppressed migration and invasion of murine breast cancer cells by a mechanism involving inhibition of the phosphoinositide 3‐kinase/Akt pathway [86]. In viral oncogene Jun‐transformed cells, however, re‐expression of GM3 correlated with a reduced invasive ability [87] (Table 1). Actually, an enhanced synthesis of GM3 allowed for the reversion of the Jun‐induced oncogenic phenotype [87]. Likewise, exogenously applied GM3 not only inhibited proliferation and invasiveness of glioma cells but also significantly prolonged survival of rats with meningeal gliomatosis [88]. A comparable therapeutic effect of ganglioside GM3 was also obtained in bladder cancer cells and in a mouse model of orthotopic bladder cancer [89]. These results indicate that GM3 not only blocks cell invasion but also affects cell–cell adhesion and induces apoptosis. On the other hand, de‐*N‐*acetyl GM3, a GM3 derivative lacking the acetyl group of sialic acid, was found to be highly expressed in human melanoma cells and critical for their invasive potential [90]. Together, these studies clearly reflect the contrasting effects of ganglioside GM3 on the invasive potential of different cancer cells. Interestingly, data regarding the effect of GM2, a direct descendant of GM3 (Fig. 1), on tumor invasiveness and malignancy are quite consistent. It has been long known that GM2, which is rather weakly expressed in normal tissue, is highly abundant in several human malignant tumors, including melanomas, gliomas, and neuroblastomas [91]. Recent studies confirm that GM2 induces invasiveness in irradiation‐tolerant human lung adenocarcinoma cells [92] and promotes invasion and hence malignancy of human pancreatic ductal adenocarcinoma cells [93]. In contrast to ganglioside GM2, its direct descendent GM1 was shown to exert anticancerous effects. Thus, reduction of GM1 expression increased proliferation and invasion of lung cancer cells, making them highly metastatic [94]. Since these gangliosides are metabolically closely linked, one possible explanation for these conflicting findings is that the manipulation of one ganglioside can also cause changes in the relative abundance of its close relatives. How manipulating GM3, for example, can in turn affect the abundance of GM2 and GM1 in different cell types could provide valuable insight into the true function of these sphingolipids in cancer progression.

A similarly dualistic role can also be observed with disialo‐gangliosides (GDs). Thus, GD2/GD3‐positive human osteosarcoma cell lines are far more invasive than their GD2/GD3‐negative counterparts [95], and GD3 was also found to be required for the invasion of malignant melanomas [96]. Consistently, inhibition of GD3 and GD2 synthesis strongly inhibited the invasiveness of human lung cancer cell lines [97]. On the other hand, overexpression of GD3 synthase (sialyltransferase II; SATII; Fig. 1), thereby increasing the concentration of GD3 in the plasma membrane, strongly inhibited the invasiveness of a human breast cancer cell line [98] (Table 1). Also, the expression of the more complex ganglioside GD1a was directly correlated with the invasiveness of cancer cells: The higher its expression, the lower the metastatic potential [99]. Consistently, treatment of a highly metastatic osteosarcoma cell line with GD1a severely impeded its migration capabilities [100]. One possible explanation for these seemingly conflicting roles of gangliosides in cancer cell invasion is that they do not exist in a vacuum, but rather their effects on cell shape and motility are dependent on the other lipids that are present in the cell membrane. For example, one study found that remodeling of lipid rafts in a melanoma cell line by increasing GD1b, GT1b, and GM1a while reducing GM2, GM3, GD2, and GD3 significantly suppressed cell growth and invasion [101]. Another study suggested that exogenously supplied as well as cell surface gangliosides prevent glioma cell invasion based on their adhesion‐promoting action to basement membrane components [102].

Together, these studies show the complexity of the effects of gangliosides on the invasive potential of transformed cells. They indicate, moreover, that the plasma membrane has to contain the right balance of lipids and receptors in order for the appropriate changes in cell shape to occur for tissue invasion.

## 4 Step 3: Angiogenesis

Once the cancerous mass reaches a certain size, the cells need to find a way to supply themselves with oxygen and nutrients in order to survive. To do this, cancer cells take advantage of processes usually reserved for development, growth, and wound healing, by forming new blood vessels that can bring the resources they need toward the center of the newly formed mass [13].

As an intercellular signaling molecule with a role in embryogenesis, S1P is also involved in the formation of new blood vessels during embryonic development [103], and cancerous cells can also appropriate this function to their own ends. S1P has been found to be a key modulator of angiogenesis in mouse models of breast cancer [104] and in human diffuse large B‐cell lymphoma [105], ovarian cancer [106], liver cancer [107], and glioblastoma [108]. Furthermore, treatment of a mouse model of melanoma with the immunomodulator FTY720 inhibited tumor growth and angiogenesis by internalizing S1P receptor 1 (S1P_1_) and desensitizing it to S1P [109]. In a human *in vitro* model of liver cancer, the downregulation of SK1 by miR‐506 inhibited S1P production and thus tumor angiogenesis [110]. Consistently, miR‐506 was also found to inhibit angiogenesis in human gastric cancer, though whether this was due to S1P inhibition was not explored [111].

As an intercellular signaling molecule, S1P is dependent on certain receptors on the cell membrane in order to trigger the appropriate signaling cascades and initiate processes such as invasion or angiogenesis. Without those receptors, S1P will not be able to complete its task as an intercellular messenger. Interestingly, some studies suggest that different S1P receptors can trigger opposing processes when in the presence of S1P, indicating that the abundance of specific receptors on the cell membrane is just as important as the presence of the extracellular messenger itself. For example, while most of the aforementioned studies focused on the signaling of S1P via the receptor S1PR_1_, a study in mice found that the receptor S1PR_2_ actually triggers a potent anti‐angiogenic response [112] (Table 1). Consistently, blocking S1PR_2_ with the antagonist JTE‐013 enhanced cell migration and angiogenesis of mouse vascular endothelial cells [113]. However, in a study on human neuroblastoma, JTE‐013‐mediated inhibition of S1PR_2_ actually blocked angiogenesis and tumor growth [114]. These studies highlight how extracellular messengers and receptors interact and elicit responses based on numerous, interplaying factors. Also, on top of that, certain gangliosides, such as GD3 or GM2, were reported to promote tumor‐associated angiogenesis [115].

Tumor cells communicate and coordinate with one another not only with the use of intercellular messengers such as S1P, but also via the secretion of exosomes into the extracellular milieu. The secretory machinery required for the packaging of these exosomes is dependent on the generation of ceramide in the plasma membrane by neutral sphingomyelinase 2 (nSMase2; Fig. 2) [116], and, in a mouse breast cancer cell line, the ceramide‐dependent production of exosomes was required for tumor angiogenesis [117]. Furthermore, a study in breast cancer cells indicated that these exosomes also contain Globo‐H ceramide, which, when incorporated into neighboring cells, greatly augmented tumor angiogenesis [118] (Table 1). Of interest, the tumor‐associated antigen Globo‐H hexasaccharide linked to ceramide [Fucα(1‐2)Galβ(1‐3)GalNAcβ(1‐3)Galα(1‐4)Galβ(1‐4)Glc‐β‐ceramide] is the result of aberrant glycosylation, a characteristic event observed during carcinogenesis.

## 5 Step 4: Metastasis

If a tumor wishes to spread throughout the organism, it must first gain the ability to metastasize. This process cannot be completed without the acquisition of two new abilities: First, the cancerous cells must be able to survive the detachment from the extracellular matrix. Second, they must be able to attach themselves to another tissue, where they can begin anew the processes of proliferation, invasion, and angiogenesis [119].

Solid cancers shed circulating tumor cells (CTCs) in the form of single or clustered cells, and the latter display an amazing ability to initiate metastasis [120]. The biological mechanism underlying the shedding of CTC clusters from a primary tumor is largely unknown. In a very recent study, the authors convincingly demonstrated that the majority of CTC clusters are undergoing hypoxia, whereas single CTCs are largely normoxic. Thus, inhibition of tumor vascularization and hence intratumor hypoxia leads to the shrinkage of the primary tumor but simultaneously to the formation of clustered CTCs with high metastatic ability, whereas a pro‐angiogenic therapy favors the tumor growth but suppresses metastatic potential through prevention of CTC cluster generation [120].

As with tumor invasion, metastasis also requires the cell to undergo structural as well as signaling changes. As important components of the plasma membrane, gangliosides strongly regulate cell adhesion/motility and thus initiate tumor metastasis [115]. Hence, specific gangliosides are associated with certain kinds of cancer and have therefore been proposed as potential biomarkers. In a study of breast cancer patients, GM3 was found to be an excellent diagnostic marker for the luminal B breast cancer subtype [121], whereas GM2 has been proposed as a potential biomarker for cholangiocarcinoma [122]. In children with neuroblastoma, GD2 shed from tumors into the circulation was found to be a highly significant predictor of high‐risk tumors [123], while the ganglioside profile of patient‐derived melanoma cells was predictive of aggressiveness [124] and survival [125].

Although biomarker studies are by definition correlative, there have also been studies implicating gangliosides as having a causative role in tumor metastasis. GalNAc‐DSLc4, a ganglioside with a hybrid structure between the ganglio‐ and the lacto‐series (Fig. 1, insert) isolated from a renal carcinoma cell line [126], was found to recruit integrin β1 to lipid rafts, where they cooperate to adhere to metastatic sites, potentially also implicating GalNAc‐DSLc4 in the promotion of distant metastasis [127]. In a murine lymphoma model, treatment with a monoclonal antibody against GD2 was sufficient to suppress tumor micrometastasis [128], while anti‐GM2 antibodies inhibited multiple organ metastasis of small‐cell lung cancer [129] (Table 1). A more recent study showed that diverse gangliosides differentially regulate cytoskeleton and signaling molecules and hence have distinct functions in tumor progression [130]. They compared the malignancy of the melanoma‐associated gangliosides GD3 and GD2 [131, 132] with that of other gangliosides, including GM3, GM2, and GM1. Only GD3 and GD2 stimulated AKT phosphorylation and hence cell growth. Also, both GD3 and GD2 colocalized at the leading edges of cells with F‐actin. However, their effect on invasive potential differed significantly. GD3+ cells showed increased migration and invasion, whereas GD2+ showed substantially increased adhesion and spreading [130]. Consistently, the tyrosine phosphorylation level of p130CAS, a component of the integrin machinery involved in cell motility [133], as well as of focal adhesion kinase and of paxillin, was augmented in GD3+ cells [96, 134] but not in GD2+ cells [130]. In a human malignant melanoma cell line, the colocalization of GD3 with integrin β1 in lipid rafts on the plasma membrane was implicated in increased integrin‐mediated adhesion and signaling often indicative for malignant transformation [135]. Apparently, GD3 is persistently expressed during tumor development, expansion, and invasion into surrounding tissues, leading to metastasis, whereas GD2 is mainly expressed at the later stage, conferring solid adhesion and fixation at the metastasized sites [130]. Taken together, these studies suggest that gangliosides are not only correlatively associated with multiple types of cancer, but that they also have an active role to play in the metastatic potential of tumorigenic cells.

As there is some overlap in the signaling cascades involved in tumor invasion and metastasis, it is unsurprising that S1P has also been implicated in the latter. As already mentioned above, SK1, catalyzing the second step in the production of S1P from ceramide, was found to be overexpressed in patient‐derived breast cancer cells. In experiments in nude mice, *Sphk1* expression was found to be a key regulator in either promoting or inhibiting spontaneous metastasis to the lungs by controlling the expression of the metastasis‐promoting gene fascin actin‐bundling protein 1 (*FSCN1*) [136]. Consistently, a study of prostate and bladder cancer cells found that systemic S1P regulated metastasis to the lungs, and genetic loss of *Sphk1* suppressed this phenotype by activating the metastasis suppressor gene *Brms1* [137]. Thus, SK1 upregulation is often associated with poor prognosis and increased cancer metastasis [136, 138, 139, 140].

Anoikis is a cell death program usually activated when cells detach from the extracellular matrix. In order for tumorigenic cells to metastasize, they must be able to shut down this program in order to survive this detachment and travel through the organism to sites at which they can form new colonies. While ceramides have been the focus of many cancer studies due to their professed pro‐apoptotic function, there is some evidence that they can also promote anoikis. Ceramides have been shown to be powerful activators of death‐associated protein kinase (DAPK), a strong tumor suppressor and inducer of anoikis [141]. Treatment of both pancreatic and breast cancer cell lines with nanoliposomes containing C_6_‐ceramide resulted in metastasis suppression by inducing anoikis [142]. Another study in melanoma and breast cancer cells treated with C_6_‐ceramide nanoliposomes also found a suppression of metastasis, though whether anoikis was involved was not investigated [143] (Table 1).

Despite evidence that ceramides are suppressors of metastasis, other studies have found elevated ceramide levels in cancer patients. C_16_‐ and C_24_‐ceramides were found to be elevated in both human head and neck squamous cell carcinoma [144] and breast tumor biopsies when compared to benign controls, and C_16_‐ceramide was even associated with metastasis [145]. While these studies remain correlative, it is worth asking why a supposedly pro‐apoptotic family of bioactive lipids should also be biomarkers for metastatic carcinoma. Further studies in this field that dissect the distinct roles of different ceramides in cancer will certainly help shed light on this dilemma.

## 6 Step 5: Immune response

Even if a tumorigenic cell succeeds in suppressing anoikis so that it can travel to another site, it can still encounter immune cells that are programmed to identify and destroy transformed cells. In order to successfully complete metastasis, therefore, cancer cells must evade these immune cells on patrol.

As mentioned above, tumor‐derived gangliosides are often useful oncogenic biomarkers. However, studies from the 1980s showed that gangliosides are also potent inhibitors of the cellular immune response [146, 147]. Based on these findings, it was hypothesized that tumor‐derived gangliosides are able to abrogate the host antitumor immune response, thus promoting tumorigenicity. Indeed, a few years later a correlation between ganglioside shedding and tumorigenicity was established [148]. Moreover, in patients with neuroblastoma, the shedding of high GD2 levels at diagnosis could be correlated with accelerated tumor progression [149] and has also been well documented in small‐cell lung cancers, melanoma, and osteosarcomas [150, 151, 152]. It is therefore not surprising that GD2‐specific monoclonal antibodies were extensively tested in clinical trials [153] and are used as lead compounds for continuous improvement [152, 154].

While clinical studies tackle the question as to whether or not gangliosides can have a causative as well as a correlative role in tumor progression, other studies have demonstrated that some cerebrosides can affect the immune response to cancer. Natural killer T (NKT) cells are a subpopulation of lymphocytes whose job is to seek and destroy transformed cells, such as metastasizing tumors. Studies have shown that, when presented with the cerebroside α‐galactosylceramide (α‐GalCer), *in vivo* populations of NKT cells rapidly expand. This expansion leads to a powerful suppression of metastasis in mice and was sufficient to completely abolish the spread of B16 melanoma cells to the liver [155]. A similar study found that injection of dendritic cells loaded with α‐GalCer also led to a rapid NKT cell expansion in humans [156] (Table 1). Given that this treatment was found to be well tolerated even in very sick patients [157], clinical trials are still ongoing to determine its efficacy in treating non‐small‐cell lung cancer [158]. These studies elucidate an interesting role for α‐GalCer in the immune response to metastatic cells.

As indicated in ‘Step 2: Invasion’, S1P signaling via its receptors has long been known to perform a critical role in immune cell motility and differentiation [64, 159]. It is therefore not surprising that a growing body of evidence indicates that modulation of S1P and its receptors regulates the efficacy of the immune system in its targeting of tumorigenic cells. Thus, both S1P concentration and S1PR_1_ expression are essential for the egress of lymphocytes and naïve human T cells from the thymus and lymph nodes to peripheral tissues [160, 161, 162]. Exit of lymphocytes from lymph nodes also is controlled by S1P released into the lymph by lymphatic endothelial cells [163]. Although deletion of systemic SK1/S1P was reported to suppress lung metastasis [137], the role of the immune system in this process was answered only years later by a genome‐wide *in vivo* screen that identified S1P transporter spinster homolog 2 (*Spns2*) as the missing link between immune response and lung colonization by cancer cells [164]. Global or lymphatic endothelial‐specific deletion of *Spns2* resulted in lymphopenia and provoked a much higher percentage of effector T cells and NKT cells, which in turn led to a more effective targeting of tumorigenic cells and a suppression of metastasis [164]. In bladder cancer, on the other hand, the increased expression of S1PR_1_ in cancer cells was found to be positively correlated with the number of tumor‐infiltrated regulatory T cells (Tregs), whose role is to suppress the antitumor immune response, and hence predicted a poor prognosis for the patients [165]. Thus, the question arises: which factors define the role of S1P as friend or foe of the immune response to cancer? Given that S1P has an important role to play in healthy immune cell trafficking, it is likely that the role of S1P in immune detection or evasion is highly dependent on the microenvironment and biological context; thus, any conclusions as to its role in cancer progression should take this into consideration. The presence or even proportion of different receptors found on the cell membranes, the composition of cellular populations in any given microenvironment, the other bioactive molecules present in the extracellular milieu, or the way that cancerous cells have reprogrammed either their secretion of S1P or their response to S1P binding, all could influence the interplay between the immune system and the tumorigenic cell population.

## 7 Sphingolipids, inflammation, and cancer

When the immune system mounts an attack, it elicits what is referred to as an inflammatory response. In the context of cancer, increased levels of inflammation are often linked to the development and progression of malignant tumors [166], with sites of chronic inflammation being a common component of tumorigenic microenvironments [167]. Hence, tumor cells use inflammatory markers, including cytokines and selectins, to support them in the processes of invasion, migration, and metastasis perceived in cancer development [166]. Different factors can lead to or exacerbate chronic inflammation, with one group of molecules involved being bioactive sphingolipids. Particularly, ceramide, S1P, and C1P have been widely implicated in immune‐dependent and vascular‐related chronic inflammatory diseases due to their involvement in the regulation of cell migration, differentiation, proliferation, apoptosis, and the stress response [168, 169, 170]. For example, the metabolism of dietary and membrane sphingolipids in the intestine generates ceramide, S1P, and C1P (Fig. 1), which in turn affect growth, differentiation, and apoptosis of immunocompetent cells in the gastrointestinal tract and have been associated with the development of inflammatory bowel disease (IBD) and also of colon cancer [171].

The relationship between sphingolipids, inflammation, and cancer is, however, rather complex. Although ceramide generally acts as a powerful tumor suppressor [19], it was also reported to activate transcription factor NFκB, a key pro‐inflammatory and anti‐apoptotic molecule [172]. Similarly, C1P was initially described as pro‐inflammatory based on its ability to stimulate the release of arachidonic acid and the subsequent production of pro‐inflammatory eicosanoids in lung carcinoma cells [173]. A more recent study, however, describes C1P as a potent inhibitor of both acute and chronic airway inflammation [174]. In the same year, another study found that the systemic deletion of acid sphingomyelinase and hence a decrease of ceramide are correlated with a decreased metastatic dissemination of tumor cells in the lung [175]. The fact that C1P is generated at the expense of ceramide and was shown to inhibit the activity and expression of NFκB and of pro‐inflammatory cytokines including TNFα, IL‐1β, IL‐6, and macrophage inflammatory protein‐2 (MIP‐2) in murine lungs and human airway epithelial cells and neutrophils [174] argues for its protective role in pulmonary tumor dissemination [169]. On the other hand, C1P, known to promote macrophage migration, has been shown to also trigger migration and thus invasiveness of tumor cells [81, 83] (Table 1).

Although the role of S1P in inflammation is rather controversial, and subject of constant debate [169], its role in inflammation‐associated cancer development appears to be relatively clear. An explanation might be the fact that, in addition to its pro‐inflammatory effect, S1P signaling promotes neovascularization and proliferation while inhibiting apoptosis [176, 177, 178]. Thus, S1P/S1PR_1_ signaling was shown to be closely linked to a persistent activation of STAT3 in cancer cells [179]. STAT3 is a transcription factor for the *S1pr1* gene, and enhanced *S1pr1* expression activates STAT3 via upregulation of JAK2 tyrosine kinase activity and also upregulates *Il6* gene expression [179]. As aberrant IL‐6–JAK–STAT3 signaling is an important mechanism for cancer initiation, development, and progression [180], S1P/S1PR_1_ signaling accelerates tumor growth and metastasis via an IL‐6/JAK2‐mediated persistent activation of STAT3 [179]. This example documents the close and reciprocal dependence of S1P metabolism, inflammatory processes, and malignant tumor progression. Consistently, in colon cancer, which is often preceded by chronic inflammation, S1P concentration has been shown to be increased due to downregulated SPL [181] and overexpressed SK1 [47]. This once more indicates a link between carcinogenesis and the disturbed S1P metabolism, particularly with high concentrations of S1P. Indeed, accumulation of S1P caused by SPL deficiency was shown to promote cell transformation through pathways involving STAT3, JAK, and S1PR_1_ [41].

A careful look at the target molecules induced by ceramide, C1P, and S1P in inflammation and cancer (see also Table 1) reveals that they are rather related, if not the very same: for example, NFκB, TNFα, and IL‐6. Considering the closely interconnected metabolism of ceramide, C1P, and S1P, the question of whether the effects reported were always correctly assigned or whether, for example, the pro‐inflammatory activities of ceramide were in fact mediated by C1P or S1P and *vice versa* is justified (Fig. 1). Moreover, sphingolipids are linked via other molecules, such as ethanolamine phosphate, choline, serine, and fatty acids, to other lipid classes, such as eicosanoids and phospholipids, which play their own role in inflammation and cancer. Constructive debates and future studies will contribute to a better understanding of the biochemical interconversions between bioactive lipids and their role in inflammation‐associated cancer development.

## 8 Resistance to treatments

While many different chemotherapeutic compounds have been discovered over the years, the ability of tumorigenic cells to acquire a resistance to these treatments remains one of the most troubling clinical problems to overcome. In this regard, many studies have not only focused on the targeting of sphingolipids as a possible therapeutic strategy, but also as a means to resensitizing tumorigenic cells to already established chemotherapeutic drugs.

As mentioned in ‘Step 1: Proliferation’, the pro‐apoptotic properties of ceramide have made it a focus of cancer treatment, whether by direct supplementation [182] or because many chemotherapies have been found to act via an increase in endogenous ceramide levels [25, 26]. Further studies have also implicated ceramide in chemoresistance. For example, one study found that overexpression of acid sphingomyelinase, resulting in the release of ceramide from sphingomyelin (Figs 1 and 2), sensitized both human and murine glioma cells to chemotherapy [183]. Another study, in patients with stage IV breast cancer, found that C16:0 ceramide was suppressed after chemotherapy by stimulating ceramide glucosyltransferase (GlcT; see also Figs 1 and 2). Restoring ceramide levels either by inhibiting GlcT or by inhibiting C2‐ceramide treatment resensitized breast cancer cells to chemotherapy [28]. Furthermore, elevated SK1 protein levels (Figs 1 and 2) correlated with poor prognosis in patients with breast cancer [139], and treatment with either an SK2 inhibitor [184] or a ceramide analog [185] restored chemosensitivity to both chemo‐ and endocrine‐therapy‐resistant breast cancer cells. Similarly, in drug‐sensitive myeloid leukemia cells, chemotherapy‐induced inhibition of SK1 activity coupled with ceramide generation, while, in chemoresistant myeloid cells, SK1 activity persisted and no ceramide was generated upon drug treatment [186]. Accordingly, chemosensitivity could be induced by treatment with cell‐permeable ceramide analogs, whereas enforced SK1 expression and activity triggered chemoresistance [186].

However, other studies have implicated ceramide as a key modulator of the acquired resistance that some cancer cells develop in response to chemotherapeutic drugs. In other models of breast cancer, treatment with doxorubicin can augment migration and invasion [187]. Later, it was shown that doxorubicin increases neutral sphingomyelinase 2 expression, which releases ceramide at the plasma membrane (Fig. 2), driving cancer cell migration and invasion [188]. These studies stress the importance of which subcellular compartment the ceramide is released from as being one of the determining factors of the bioactive function that it will carry out, as also outlined in the ‘many ceramides’ hypothesis [189].

When modulating the abundance of distinct sphingolipids by affecting their metabolism, it is always important to remember that they are interconnected; thus, affecting one enzyme will often influence the abundance of more than one member of the sphingolipid family. For example, only two enzymatic steps are needed to generate S1P from ceramide and *vice versa* (Fig. 1). This further complicates our understanding of the effects of ceramide, as S1P has its own role to play in chemoresistance. For example, studies in lung cancer [190], ovarian cancer cell lines [191], and neuroblastoma [192] demonstrate that the stimulation of SK2 expression conferred chemoresistance via S1P, whereas inhibiting either SK2 or S1P receptors could abolish this effect. Mice deficient in S1PL, the enzyme which irreversibly cleaves S1P, have also been found to be resistant to chemotherapy‐induced apoptosis [60]. However, S1PL deficiency has been shown to have a very cell type‐specific effect on the abundance of different sphingolipids, which also implies that its effect on a cellular response to chemotherapy would likewise depend on the cell type in question. For example, in neurons, S1PL deficiency results only in accumulation of S1P and sphingosine, whereas, in MEFs, it also leads to an accumulation of ceramide, GlcCer, and ganglioside GM3 [61]. These studies further underline the importance of proceeding with caution when attempting to modulate sphingolipids by affecting their metabolic enzymes, as a disruption of the balance between recycling and *de novo* synthesis can have unforeseen consequences in different cellular environments.

As mentioned previously, gangliosides found on the plasma membrane have been implicated in oncogenic signaling [193, 194] and thus have been proposed to be useful targets for cancer treatment [195]. While one recent study suggests that targeting O‐Acetyl‐GD2, a derivative of GD2 ganglioside in which the outer sialic acid residue is modified by an O‐acetyl ester, might be helpful in treating chemoresistant glioma cells, particularly if used in combination with other drugs [196], most studies have focused on the modulation of GM3 metabolism as a means to address chemoresistant cancer cells.

The human plasma membrane‐associated ganglioside‐specific sialidase 3 (neuraminidase 3; NEU3) is crucial in the regulation of cell surface processes and has been found to be upregulated in colon [197], kidney [198], and ovarian [199] cancer cells. The increased expression of NEU3 promoted cell motility and suppressed apoptosis, thus augmenting tumor malignancy. Glycolipid analysis revealed a decrease of GM3, which was degraded by NEU3 to LacCer (Fig. 1), but no changes in GD3 [197, 198]. In renal cancer cells, the decrease of GM3 was correlated with the activation of the PI3K/Akt signaling cascade [198]. Conversely, in *NEU3*‐silenced cells, drug resistance, invasive potential, and adhesion were found to be decreased, together with restored GM3 and increased GD1a levels [200]. In prostate cancer, NEU3 has also been found to be upregulated in androgen‐independent, hormone‐therapy‐resistant cancer cells but not in androgen‐sensitive cells [201]. However, forced overexpression of NEU3 induced hormone resistance in therapy‐sensitive cells. Recently, bound polyphenols from millet bran were shown to resensitize chemoresistant human colorectal cancer cells to chemotherapy by inhibiting NEU3, thus preventing GM3 degradation and increasing its amount in the plasma membrane [202].

Taken together, one might reach the conclusion that decreasing GD2 and increasing GM3 (Fig. 1) in the plasma membrane is a good strategy in the battle against chemoresistance. However, other studies may lead one to a different conclusion. For example, GM3 is abundant in the plasma membrane of both highly metastatic melanoma cells [203] and cisplatin‐ and doxorubicin‐resistant lung cancer cells [204, 205]. Modulation of GM3 amounts led to the conclusion that this ganglioside directly conferred chemoresistance to Lewis lung carcinoma cells [205]. Once again, these studies together show that affecting the balance of different sphingolipids is a better determinant of how a cell population will behave than is the abundance of any one member of the sphingolipid family. While remaining technically challenging, a more comprehensive picture of relative sphingolipid abundance is what is often needed to unravel and fully understand the cell's response to different chemotherapeutic compounds. For example, what other sphingolipids are affected upon downregulation of NEU3, not just on the plasma membrane, but within the cell? Is it possible that it is not only the chain length or subcellular compartment of ceramide which determines its function, but also the relative abundance of S1P? Further studies are needed to see whether this is in fact what is behind these apparently conflicting roles of certain sphingolipids in cancer treatment.

## 9 Conclusions and perspectives

In this Review, we highlight the dual role of sphingolipids in cancer and how different tumors can either use sphingolipids to their advantage or find their progression blocked by their presence. While it is impossible for any one study to take every molecular event or response into account, we wish to emphasize that the greater biological context is key to not only understanding cancer progression, but also in predicting how tumors or even patients might respond to treatment with any one of these bioactive lipids. The interplay between different cellular populations, the readiness with which these cells might metabolize or transform one sphingolipid into another, the composition of lipid rafts and the presence of receptors in the cell membrane, can all influence how cells will respond to the presence of sphingolipids.

Dysregulation of sphingolipid metabolism has been demonstrated in several cancers, representing an important step in tumorigenesis from initiation and progression to host immune response. In this context, we strongly recommend an excellent review on the role of sphingolipid metabolism in melanoma progression and immune response [206]. Despite the multitude of aspects reported in the present Review, which sometimes appear conflicting, we feel that there are congruent key findings that warrant increased awareness, particularly of the larger biological context in which these bioactive sphingolipids find themselves.

The cell membrane and its components, including gangliosides in particular, open the door for the development of new therapeutic strategies against cancer [193, 207]. Thus, the high and stable presence of GD2 on cancer cells in neuroblastoma and limited expression on relevant normal tissues permits diagnosis and detection of metastases, but also treatment monitoring and, most importantly, targeting of the tumor itself. Note that therapeutic targeting of ganglioside GD2 is most advanced in neuroblastoma, the most common extracranial tumor in childhood [154]. Moreover, gangliosides GD1a and GM1a were proposed as potential improvers of the chemopreventive and/or therapeutic efficacy against human colon cancer [208]. But gangliosides are more than membrane components that modulate signaling and skeleton proteins, as several of their metabolic intermediates emerged as critical regulators of cellular processes with direct relevance to cancer, including growth, differentiation, apoptosis, and senescence [209]. It is therefore not surprising that dysregulation of their metabolism can affect the tumorigenic potential of numerous cell types. As shown here, targeting of enzymes, especially SK1, SPL, ceramide kinases, and GlcCer‐synthase, is a promising tool to overcome cancer progression.

Much has been achieved over many decades of research, but there is also a huge effort ahead of us. Thus, several molecular mechanisms regarding the mode of action specifically of S1P–S1PR signaling on proliferation, neovascularization, and inflammation have been uncovered, but there are still many open questions regarding, for example, the contrasting effects of ceramides of different chain length in diverse cancers. Also, can the host response to the tumor be improved by counteracting the generation and shedding of certain gangliosides? It is possible that, given these complex, context‐dependent roles in cancer progression, future therapies will include a certain amount of cellular characterization in order to ensure a successful outcome. Future studies will find answers to these and other questions regarding sphingolipid metabolism and cancer. However, the targeting of sphingolipid metabolism remains a promising strategy to overcome cancer.

## Conflict of interest

The authors declare no conflict of interest.

## Author contributions

AP and GvE‐D wrote the manuscript. SYA helped to prepare the figures and table.
